# Super-resolution ultrasound imaging method for microvasculature *in vivo* with a high temporal accuracy

**DOI:** 10.1038/s41598-018-32235-2

**Published:** 2018-09-17

**Authors:** Jaesok Yu, Linda Lavery, Kang Kim

**Affiliations:** 1 Center for Ultrasound Molecular Imaging and Therapeutics, Department of Medicine and Heart and Vascular Institute, University of Pittsburgh School of Medicine and University of Pittsburgh Medical Center (UPMC), Pittsburgh, PA 15261 USA; 2 Department of Bioengineering, School of Engineering, University of Pittsburgh, Pittsburgh, PA 15261 USA; 3 Division of Cardiology, Department of Medicine, University of Pittsburgh School of Medicine, Pittsburgh, PA 15261 USA; 4 McGowan Institute of Regenerative Medicine, University of Pittsburgh and University of Pittsburgh Medical Center (UPMC), Pittsburgh, PA 15219 USA

## Abstract

Traditional ultrasound imaging techniques are limited in spatial resolution to visualize angiogenic vasa vasorum that is considered as an important marker for atherosclerotic plaque progression and vulnerability. The recently introduced super-resolution imaging technique based on microbubble center localization has shown potential to achieve unprecedented high spatial resolution beyond the acoustic diffraction limit. However, a major drawback of the current super-resolution imaging approach is low temporal resolution because it requires a large number of imaging frames. In this study, a new imaging sequence and signal processing approach for super-resolution ultrasound imaging are presented to improve temporal resolution by employing deconvolution and spatio-temporal-interframe-correlation based data acquisition. *In vivo* feasibility of the developed technology is demonstrated and evaluated in imaging vasa vasorum in the rabbit atherosclerosis model. The proposed method not only identifies a tiny vessel with a diameter of 41 μm, 5 times higher spatial resolution than the acoustic diffraction limit at 7.7 MHz, but also significantly improves temporal resolution that allows for imaging vessels over cardiac motion.

## Introduction

Acute coronary syndromes (ACS), a leading cause of morbidity and mortality in the US and Europe, is generally caused by the plaque rupture or erosion^1–4^. Extensive efforts to characterize and predict vulnerable plaques assessing a few known markers have been made for several decades^1–7^. It has been found that abnormally dense neovascularization of the vessel wall, often infiltrating into the plaque core, is associated with development of atherosclerotic plaque and progression of the disease^4,8^. These micro-vasculatures may lead to intraplaque hemorrhage, which typically accompanies unstable plaque^4,8,9^. Therefore, abnormal proliferation of adventitial vasa vasorum (VV) is an important clinical imaging target to assess vulnerability of the atherosclerotic plaques^4–8,10^. However, the lack of adequate noninvasive and high-resolution imaging technology to visualize VV is a big challenge. Micro-CT, optical coherent tomography (OCT), intravascular ultrasound (IVUS) angiography and contrast-enhanced ultrasound (CEU) imaging have been demonstrated to image VV in pre-clinical studies, but these technologies have suffered from hazardous radiation (micro-CT), poor imaging depth (OCT), invasive approach (IVUS) and insufficient spatial resolution (CEU)^5,6,11–16^.

CEU is one of the well-established imaging modalities to evaluate microvasculature *in vivo* animal study using gas-filled microbubbles that can provide high echogenic contrast^6,13,17^. In previous studies with CEU using a mid-frequency transcutaneous linear array transducer (Transmit at 7 MHz and receive at 15 MHz), they have shown the correlation of adventitial VV density based on ultrasonic image intensity with VV progression^6,13,17^. However, identifying individual VV, especially those near the lumen of the main vessel, was limited mainly due to low spatial resolution at mid-frequency^6^. Because of this limitation with spatial resolution, the accuracy of VV density measure therefore was limited. High spatial resolution is essential for separating individual tiny VV from the lumen of the main vessel to improve the accuracy of adventitial VV density as an indicator of VV progression. A high-frequency transcutaneous linear array transducer centered at 40 MHz has shown its spatial resolution as high as 67 µm in a tissue-mimicking phantom but with a limited imaging depth less than 14 mm^18^. A high-frequency single element transducer ranging 30–150 MHz is preferably utilized in IVUS. However, conventional (30–50 MHz) and high frequency (90–150 MHz) IVUS are limited to the spatial resolution (>100 µm) and the imaging depth (<2 mm), respectively^19^. Recently, multi-frequency IVUS (or super-harmonic IVUS) has been widely studied to maintain the advantage of both deep imaging depth (>2.5 mm) and improved spatial resolution (<50 µm) by incorporating relatively low frequency transmit (30–50 MHz) and high frequency receive (90–150 MHz)^19–22^. However, this technology was only evaluated *in vitro* or *ex vivo* so far, therefore *in vivo* study should be followed. In addition, IVUS is not ideal if considering eventual translation with full noninvasiveness. Therefore, transcutaneous imaging approach using a mid-frequency linear array transducer commonly used in the clinic would be sought after.

Super-resolution US imaging technology has been recently introduced to overcome the limitation of inherent spatial resolution of US imaging defined by the acoustic diffraction limit^23–25^. This approach utilizes two state-of-the-art technologies; tissue rejection and microbubbles localization technique^24,26–28^. It is known that ultrafast plane wave imaging in general significantly improves the performance of the eigen decomposition based adaptive clutter filtering technique, outperforming in suppressing stationary signal that comes from the clutters, compared to conventional clutter filtering techniques used in typical Doppler imaging^26^. Moreover, microbubble center localization technique allows to estimate each location of microbubbles in sub-pixel level precision^24,27,28^. Each microbubble could be considered as a point source because its size (~3 µm) is much smaller than the spatial resolution of the imaging system that operates at mid-frequency ultrasound of around 5–10 MHz (100~200 µm). Therefore, received echo signal from individual microbubble would be represented as a point spread function (PSF) of the imaging system, and each microbubble is expected to be located at the centroid of the PSF. After summing up localized microbubbles in blood flow at different times over a large number of frames, a vascular network image in high spatial resolution beyond the acoustic diffraction limit can be formed. Errico *et al*., successfully reconstructed single static super-resolved image of rat brain vasculature network, identifying the micro-vessel in diameter as small as 9 μm full-width at half-maximum (FWHM) with using a 20 MHz linear array transducer^24^. However, the major drawback of this method requires a long data acquisition time of 150 seconds for a total of 75,000 frames at 500 frames per second. Requiring a long data acquisition time makes this imaging technology susceptible to motion artifacts and therefore hinders widespread of this technology for various applications.

Here, we present a new approach of super-resolution US imaging technology to achieve a high temporal resolution as well. A strategically designed approach in two steps is employed to drastically improve temporal resolution; (1) deconvolution localization technique to reduce data acquisition time, and (2) spatio-temporal-interframe-correlation (STIC) based data acquisition to compensate motion over reduced data acquisition time. First, applying deconvolution separates each center of microbubbles from densely grouped microbubbles while previous approach, 2D Gaussian fitting, typically requires discarding such frames in which clumped microbubbles cannot be separated. As such, deconvolution technique can significantly reduce data acquisition time. However, reduced data acquisition time using deconvolution only is not short enough to ignore fast physiologic motion such as cardiac-dependent motion. In addition to deconvolution technique, therefore, STIC data acquisition inspired from 3D fetal echo-cardiology imaging technique is adapted to further overcome fast physiologic motion^29^. STIC acquisition allows it to synchronize collected images over multiple cardiac cycles. *In vivo* feasibility of the developed technique in identifying VV is demonstrated in rabbit atherosclerotic plaque model.

## Results

### Deconvolution based super-resolution ultrasound imaging

**Figure 1:**
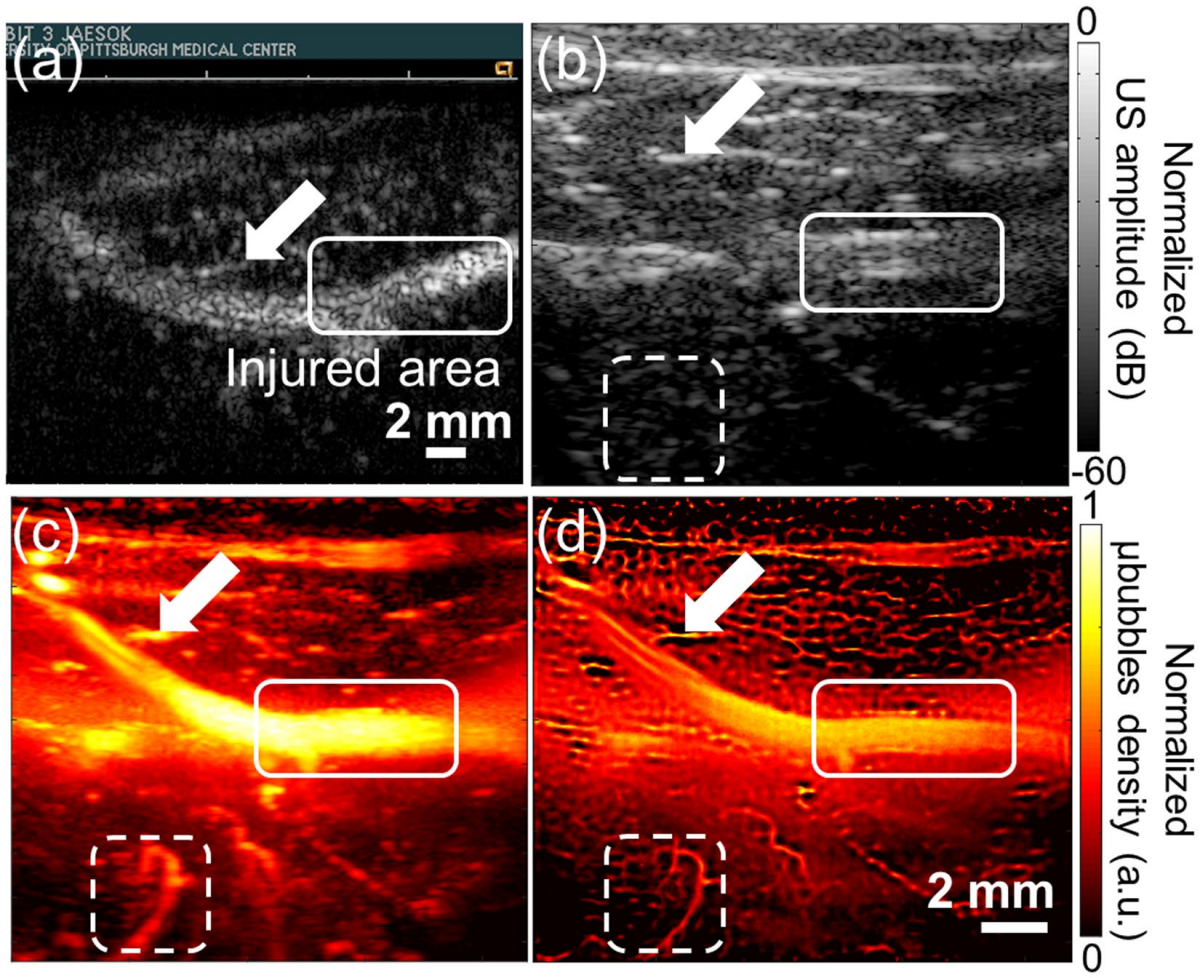
Comparison of several imaging modalities. (**a**) Cadence^TR^ contrast-enhanced imaging with microbubbles acquired by commercial ultrasound scanner (Sequoia 512, Siemens), (**b**) conventional B-mode imaging, (**b**) temporal MIP vascular imaging, (**d**) proposed super-resolution imaging of ROI. Same raw data is used to reconstruct images (**b**–**d**). The white solid rectangle represents balloon-injured area that the plaque is expected to be developed. The white arrow indicates the same vessel branch that shows a correlation of images acquired by two different ultrasound scanners. White dashed rectangle represents selected ROI used in Figs 2 and 3.

**Figure 2:**
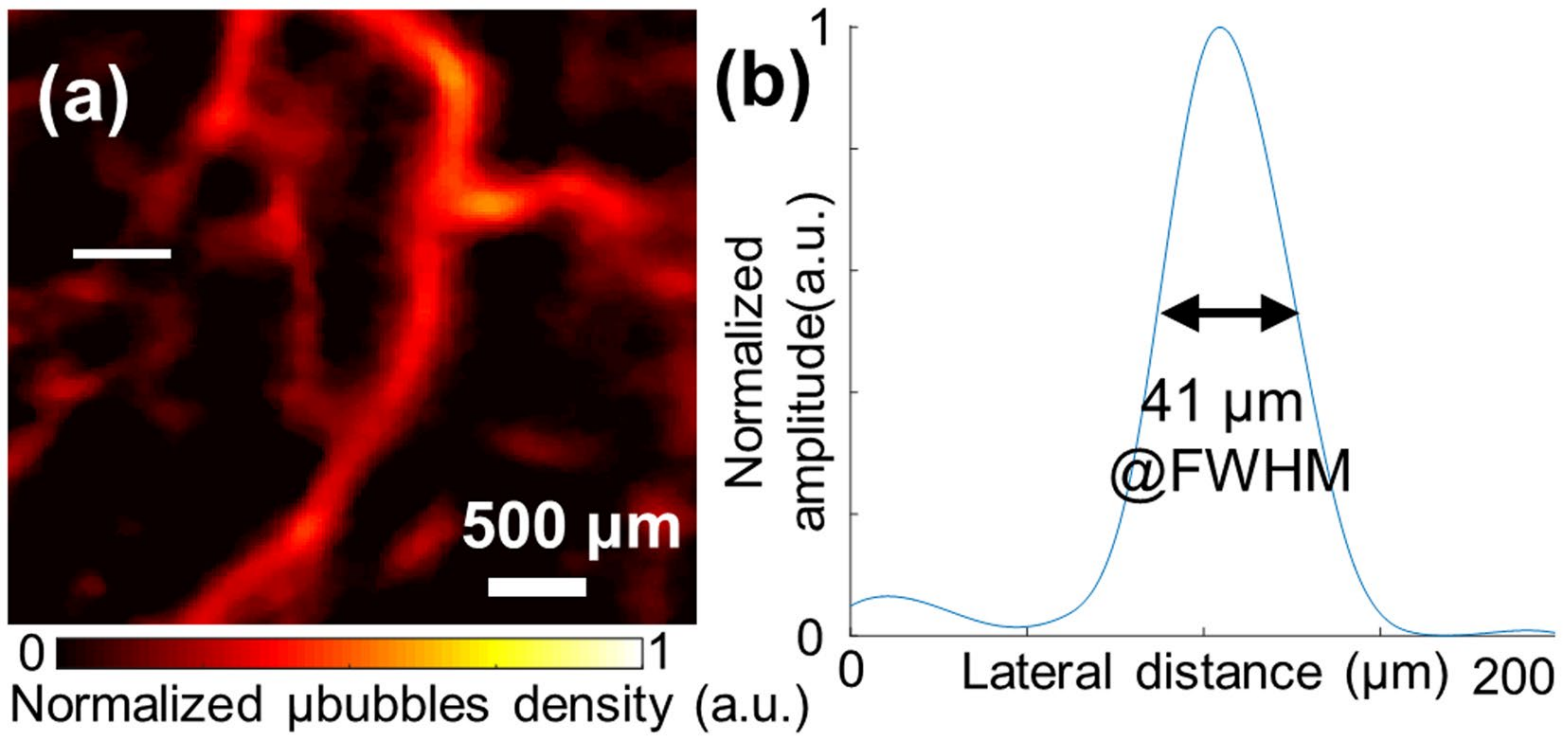
The spatial resolution of the proposed imaging method. (**a**) The detectable smallest vessel is chosen (white solid line) in the ROI of Fig. 1(b–d) indicated by the white dashed rectangle, (**b**) Spatial profile of the selected vessel. FWHM is estimated by 41 μm (<λ/5).

In Fig. 1, our developed imaging technique on a rabbit femoral artery is compared conventional imaging technologies. The white rectangle represents the balloon injured area that is expected to develop the plaque with VV, and white arrows in all images indicate a big branch near the femoral artery that is used as the landmark to match the image plane among different modalities. Cadence^TR^ harmonic contrast-enhanced imaging technique with using microbubbles operated by a commercial ultrasound scanner (Accuson Sequoia 512, SIEMENS, Mountain view, CA) shown in Fig. 1(a) is a typical standard method in the clinic to evaluate microvasculature perfusion^6,13,30,31^. However, this technique is limited in spatial resolution to identify and separate VV from the major vessel such as the femoral artery in this study. Conventional B-mode image shown in Fig. 1(b) only illustrates structural information of bifurcation area of the rabbit femoral artery and small vasculatures surrounding the femoral artery cannot be identified due to limited spatial resolution. To amplify signal from the blood, power Doppler based temporal maximum intensity persistence (MIP) imaging technique (#’s of frames = 2,000) implemented in Verasonics system is used as shown in Fig. 1(c). This method employs a tissue-rejection filter that suppresses stationary signal from tissue and extracts only signal from flowing microbubbles. However, its spatial resolution is still insufficient to clearly identify individual VV. The proposed super-resolution ultrasound imaging method implemented in Verasonics system shown in Fig. 1(d) delineates detail of microvasculature that is shown blurred in other imaging methods due to low spatial resolution that is mainly limited by diffraction limit of the operating frequency. To estimate maximum spatial resolution of our developed method, the detectable smallest vessel in the ROI is chosen among discernable vasculatures in the Fig. 2(a) (Selected region of Fig. 1(d)). FWHM on lateral axis of the selected vessel is estimated to be 41 micron that is 5-fold smaller than wavelength as shown in Fig. 2(b).

**Figure 3:**
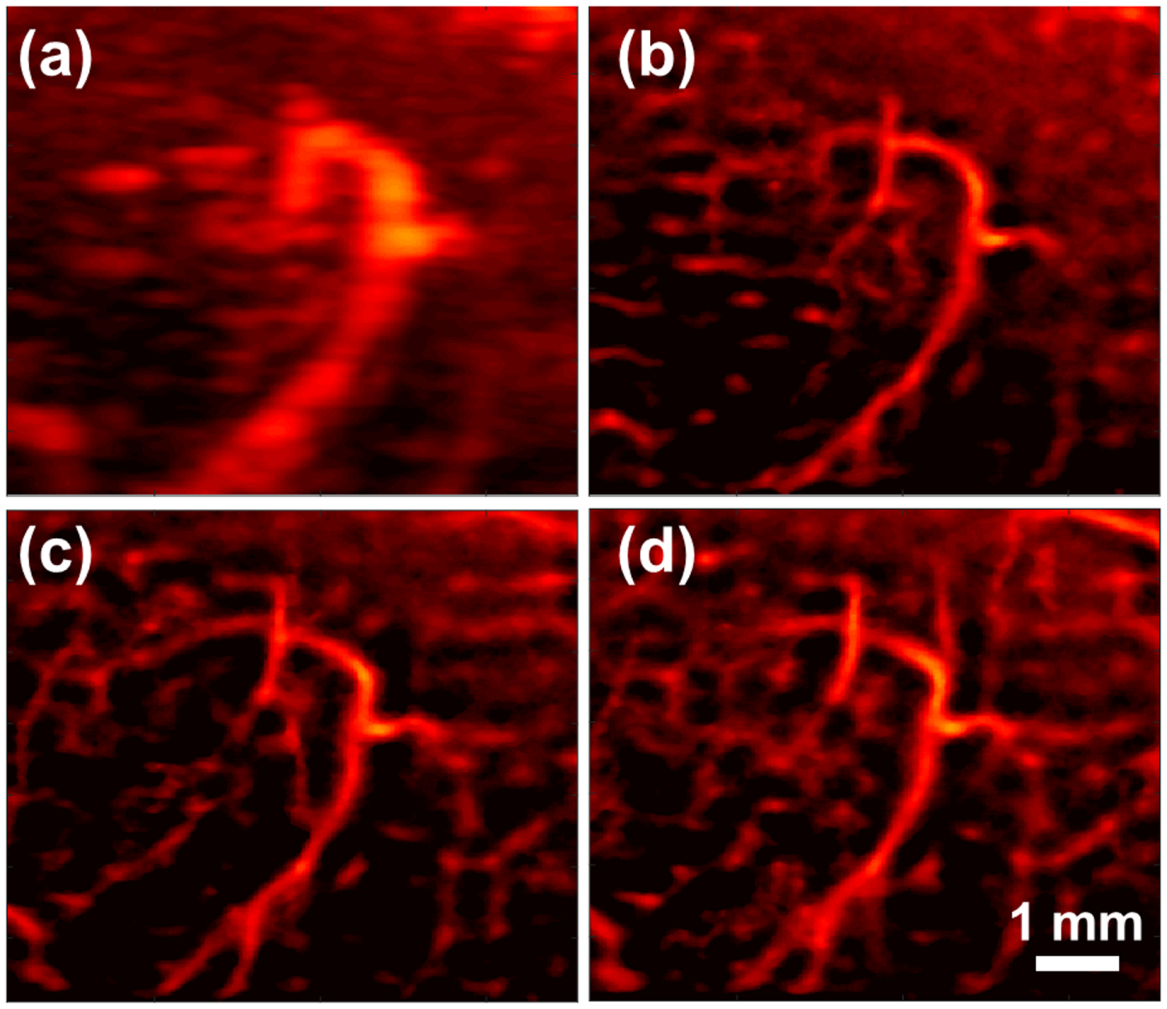
Repeatability of the proposed method. Vessel images were chosen in the ROI of Fig. 1(b–d) indicated by the white dashed rectangle (**a**) temporal MIP vascular network imaging using eigen-decomposition method. (**b**–**d**) Super-resolution images using sequentially acquired three datasets (2,000 frames per each image) from the same region of interest.

Three sequential data-set are independently acquired using our super-resolution imaging sequence to verify repeatability of our proposed method. For three independent imaging sessions, bifurcation of femoral artery and the branched vessel indicated in white arrow in Fig. 1 are identified as landmark to maintain the same imaging plane. Figure 3(a) depicts temporal MIP vascular imaging using the first data set of 2,000 frames, and Fig. 3(b–d) represents reconstructed images from sequentially acquired the first, second and third data set (2,000 frames for each acquisition), respectively using our super-resolution imaging sequence. Note that Fig. 3(a,b) are reconstructed from same raw dataset. An obviously same major vessel is observed at the center in all three reconstructed images to assure the same imaging plane among three imaging sessions. Some microvasculature in the background shown slightly different can be attributed to tilted imaging plane within three different imaging sessions.

### *In vivo* feasibility of the developed super-resolution technique in imaging vasa vasorum on a rabbit femoral artery

**Figure 4:**
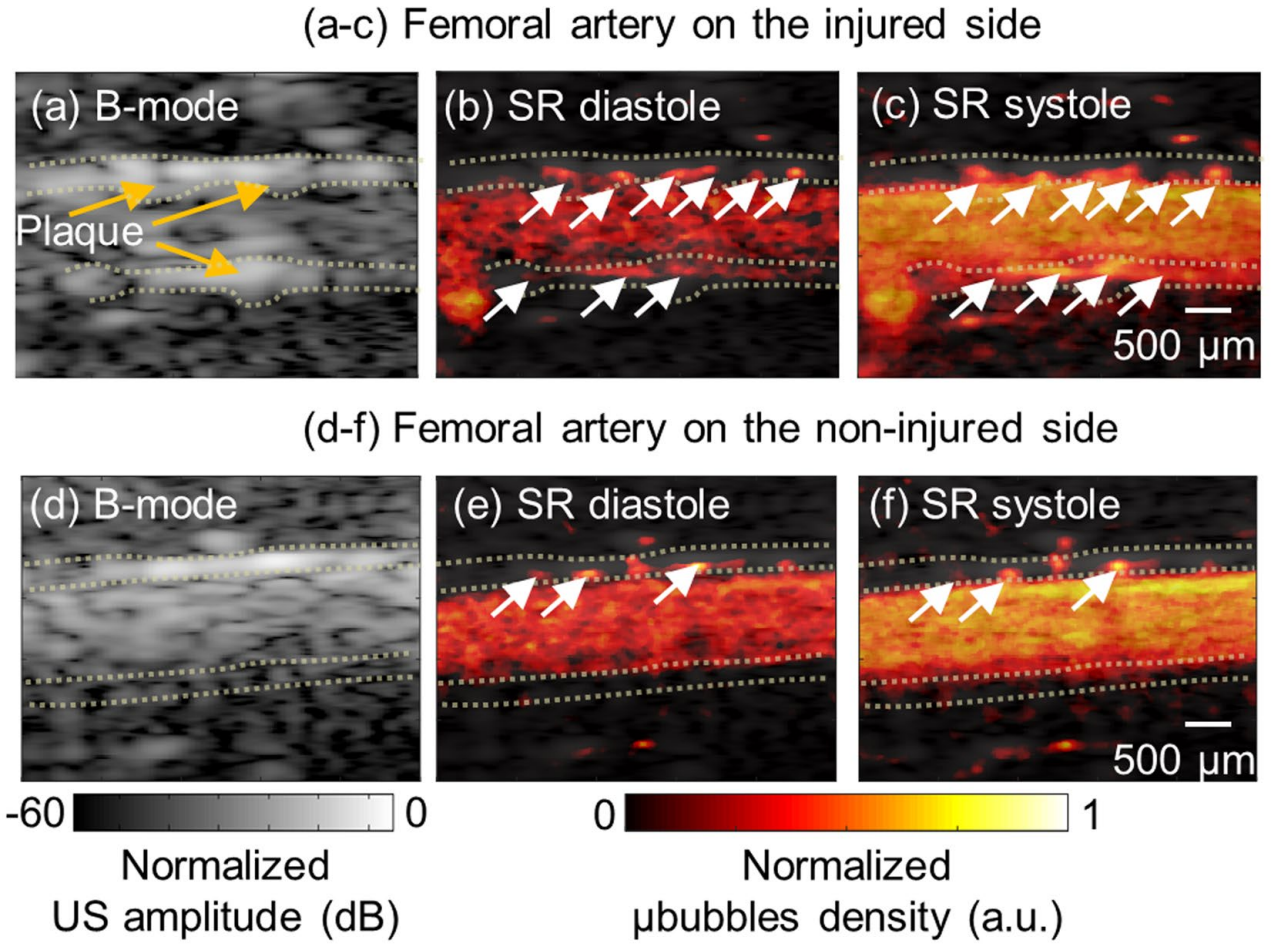
B-mode image (**a**,**d**) and corresponding super-resolution perfusion image overlaid on the B-mode image at diastole (**b**,**e**) and systole state (**c**,**f**). Top panel images are acquired from the injured side and bottom panel images are acquired from the non-injured side. Significant plaques are shown in the B-mode image on the injured side. The yellow dotted line represents adventitia region and white arrows indicate vasa vasorum in the adventitia. (Supplementary Movies 1 and 2 are available).

Conventional B-mode imaging and super resolution imaging for vascular network of both side femoral artery, injured (experimental side) and contralateral non-injured side (control side), are shown in Fig. 4. In B-mode images (Fig. 4(a,d)), adventitia area is represented in the yellow dotted line. Figure 4(b,c,e,f) show super-resolution images at diastole and systole on injured and the non-injured side, respectively. Supplementary Movies (1 and 2) for an entire cardiac cycle are provided. Each image at different stage of cardiac cycle is reconstructed from around 300 frames synchronized by STIC algorithm. The abundance of VV in adventitia indicated by white arrows can be clearly observed on the injured-side in the super-resolution ultrasound imaging. Normalized VV density on the injured-side is 0.027 ± 0.004, which is approximately 3 times greater than non-injured side with 0.010 ± 0.001. Furthermore, apparent uneven surface on the vessel wall on the injured side is observed most likely due to plaque formation on the lumen wall. The vessel wall is overall thickened (injured: 410 μm vs non-injured: 220 μm) and the lumen diameter is decreased (injured: 1.1 mm vs non-injured: 1.4 mm) in injured side. These observations are more clearly confirmed by closely looking at blood flowing through VV from adventitia into media shown in Supplementary Movies (1 and 2).

**Figure 5:**
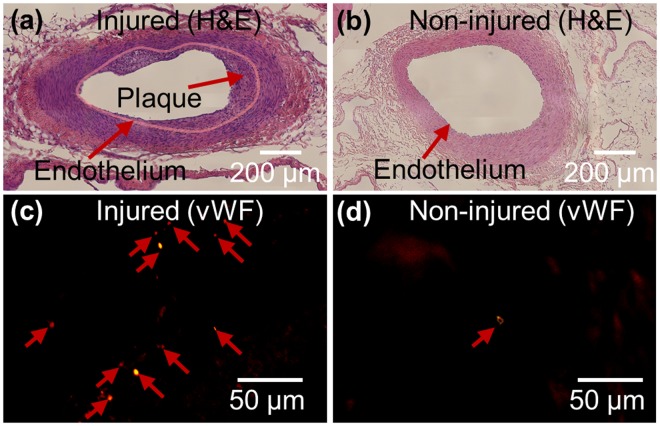
Haematoxylin and eosin stained vessel on the injured side (**a**) and non-injured side (**b**). Thirty images acquired at ×40 magnification are stitched to reconstruct an overall image of the vessel for (**a**) and (**b**). Significant plaque development is found in the injured side (**a**). Vasa vasorum on adventitia in the selected region was stained by anti-von Willebrand factor. A large number of vasa vasorums are found in adventitia on the injured side (**c**), but a few vasa vasorums are found in adventitia on the non-injured side (**d**).

VVs are more populated and believed to be infiltrated into the medial area of injured area, while less populated VV are found only in adventitial and connective tissue area in non-injured area. Atherosclerotic plaque formation and corresponding VV development is evidenced by histology of femoral artery section shown in Fig. 5. The atherosclerotic lesions are characterized on injured side by neointimal proliferation, shown in Fig. 5(b) by hematoxylin and eosin (H&E) stain and abnormally enriched VV development on adventitia, shown in Fig. 5(d) by immunofluorescence of von Willebrand Factor (vWF) stain. Pink solid line in Fig. 5(b) was drawn along endothelium in the tunica intima of the lumen. Total cholesterol level is measured 291 mg/dL that is approximately 3 times higher than standard range (<100 mg/dL) due to feeding high-fat diet.

## Discussion

It has been shown that super-resolution ultrasound imaging technique has strong potential to extend the application of ultrasound imaging with unprecedented high spatial resolution^24,25,32,33^. We further developed this imaging technology to overcome the limitation of the temporal resolution, which makes it capable of imaging of fast event. Our proposed super-resolution imaging method successfully assesses VV density on the plaque in a rabbit femoral artery with enhanced temporal resolution as well as high spatial resolution. In our approach, deconvolution and STIC data acquisition were investigated to improve temporal resolution of US sub-diffraction imaging. It should be noted that deconvolution technique is known to be sensitive to noise as discussed in method section. Thresholding used in this study can successfully suppress noise, however it potentially also suppresses true signal when signal-to-noise ratio is relatively low for example in a fast plane-wave imaging. Therefore, more advanced adaptive noise reduction approach needs to be sought after to maximize signal-to-noise ratio while maintaining high temporal resolution in the future study. In addition, STIC data acquisition is generally used to collect and synchronize 3D data acquired from fast-moving fetal heart when using a relatively slow acquisition speed. It should be noted that there is an assumption in STIC acquisition; cardiac pulsation pattern over data acquisition period is stationary. For further translation of this technique, therefore a thorough performance analysis of STIC and an adaptive algorithm development if needed for non-stationary cardiac pulsation should be ensured. With reduced total number of frames thanks to deconvolution, we were able to generate ten super-resolution images using 3,000 acquired frames that can be collected within 6 seconds. Therefore, our approach has potential for free-hand scanning in the clinics because the acquisition time is significantly shorter than free-hand data acquisition time of 3D-fetal echocardiography when using STIC acquisition in the standard clinical protocol that takes around 4~5 minutes on average^29,34^.

Since this study is concentrated on the technical development and *in vivo* feasibility demonstration, it is not intended to investigate any correlation between VV development and plaque stage with enough statistics, although this well-established rabbit model has been studied for a long time for various diseases in association with atherosclerotic plaques. Further extended study with an increased number of rabbits and time points is planned in the following study. In addition, the histology is limited for providing anatomically matched cross sections as a gold standard. An established anatomical imaging modality with high spatial resolution is necessary as a gold standard to provide more direct comparison, for example, *ex-vivo* microCT that enables to provide volumetric microvasculature information^35,36^.

## Methods

### Deconvolution-based super-resolution US imaging sequence

**Figure 6:**
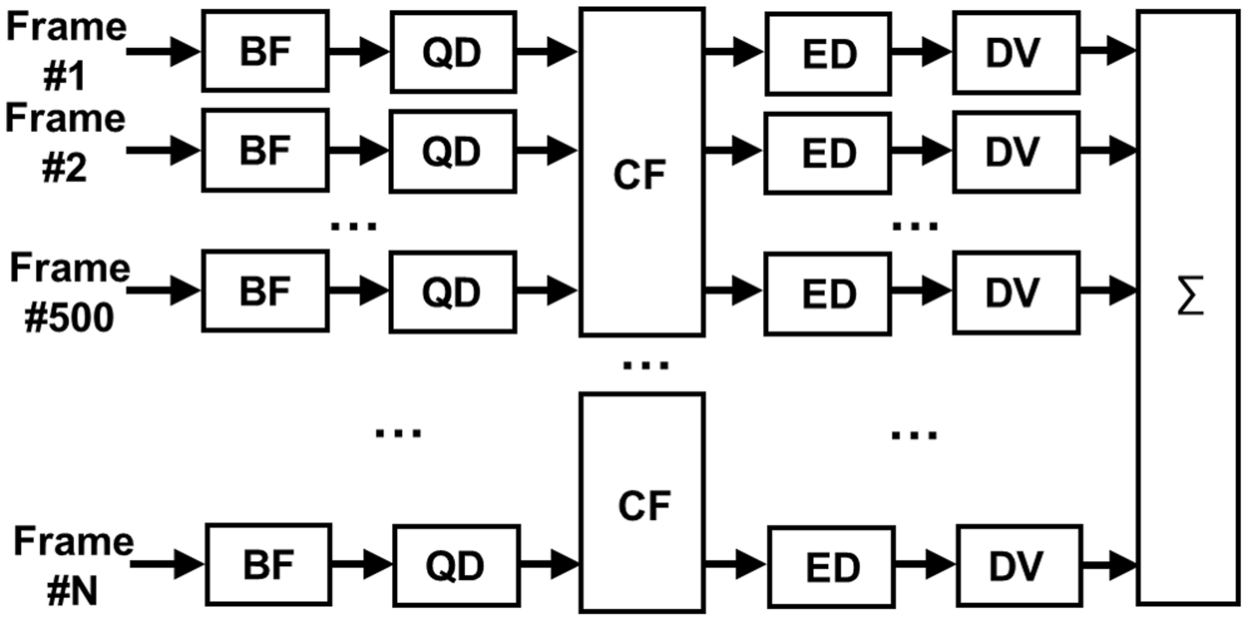
Block diagram for signal processing of super-resolution ultrasound imaging. BF: Delay-and-sum beamformer; QD: Quadrature demodulator; CF: Eigen-based spatio-temporal clutter filter; ED: Envelope detector; DV: Deconvolution with the system PSF; ∑: Integrator with STIC data alignment based on estimated cardiac pulsation.

**Figure 7:**
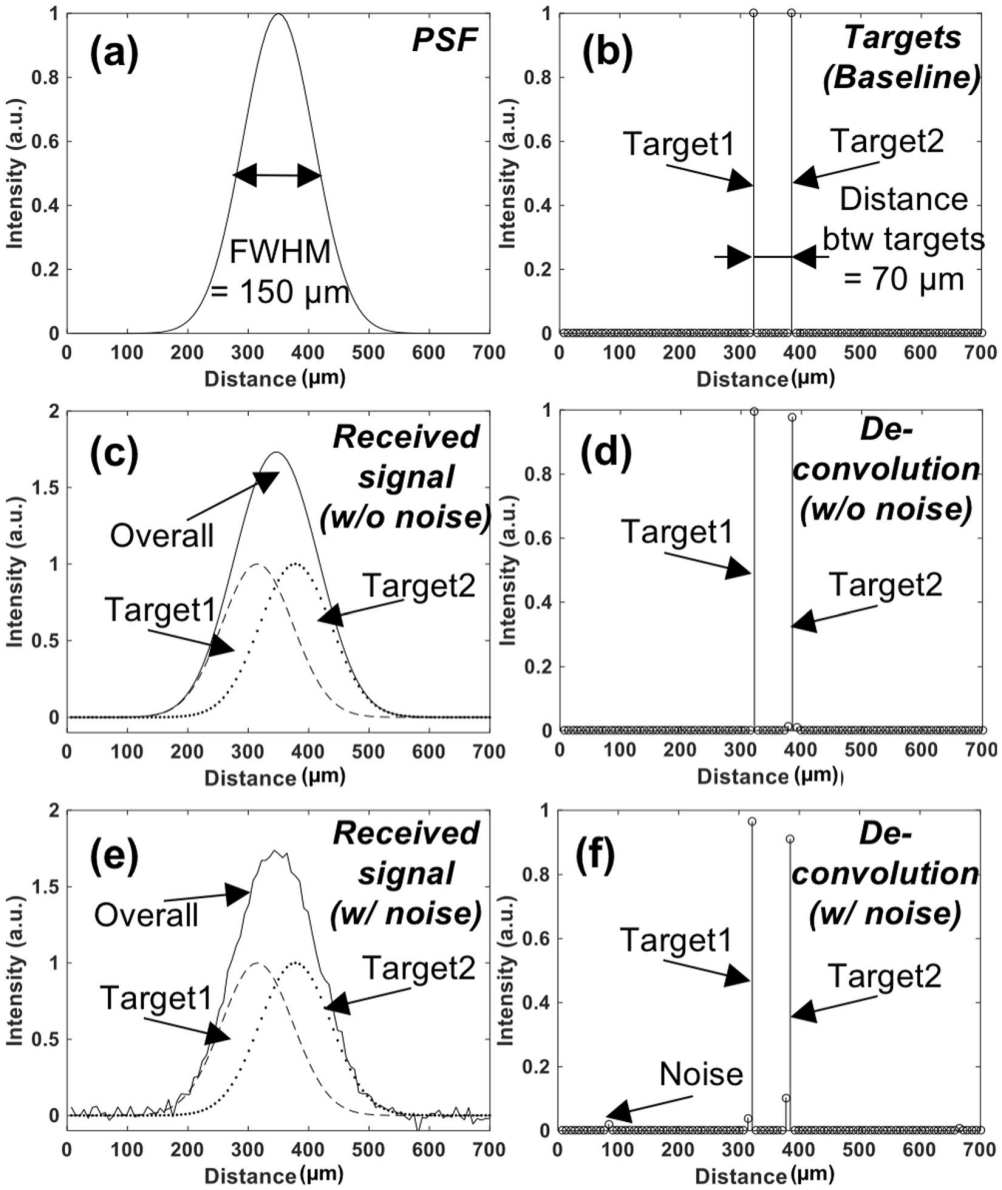
Conceptual demonstration of sub-wavelength localization using deconvolution on synthetic data. (**a**) PSF of the imaging system. FWHM is assumed as 150 µm. (**b**) The locations of the two neighboring targets (Ground truth). Two targets are positioned 70 µm apart. (**c**) The synthetic signal received from two targets is shown in (**b**) using the imaging system with PSF shown in (**a**). This signal is modeled as a received signal in the imaging system whose has PSF shown in (**a**). Two targets cannot be separated in the image due to their distance is shorter than the spatial resolution of the imaging system. (**d**) Deconvolution results of received signal shown in (**c**) using the system PSF shown in (**a**). Two targets are distinctly identified. (**e**) The synthetic signal received from two targets when noise is added is shown in (**b**) using the same imaging system. (**f**) Deconvolution results of (**e**), where two targets are clearly identified with minimal interference due to noise.

Super-resolution imaging sequence was implemented into a fully-programmable ultrasound scanner (Vantage 128, Verasonics, Kirkland, WA, USA) equipped with a mid frequency hockey stick linear array transducer (CL15-7, ATL-Philips, Bothell, WA). Ultrasound plane waves of one and half cycle at frequency of 7.7 MHz were insonified to the target with high pulse repetition frequency (PRF) of 1,500 Hz with three different steering angles (−3°, 0°, 3°) for compounding, therefore the effective frame rate is 500 Hz. Signal processing algorithm was illustrated in Fig. 6. Acquired raw radio-frequency (RF) channel data were beamformed by delay-and-sum algorithm, and then downmixed to the baseband using a quadrature demodulator. The echo signal from microbubbles were extracted from compounded analytic baseband signal of 500 frames by using a spatio-temporal eigen-based decomposition clutter filter^26,37,38^. Envelope of the echo signal from microbubbles was interpolated for an increased pixel resolution of 15 µm (lateral) × 10 µm (axial) by using the modified akima cubic hermite method and deconvolved with the measured system PSF to localize each microbubble. Richardson-Lucy (RL) deconvolution is a non-linear iterative deconvolution method that has been widely used for deblurring image with the presence of Poisson distributed noise in astronomy and biomedical applications^39–42^. The iterative process is described by

$${i}^{(k+1)}={i}^{(k)}(h\ast \frac{g}{h\,\otimes {i}^{(k)}})$$

where, * is the correlation operator, ⊗ is the convolution operator, *i*^(*k*)^ is the estimated image after *k* iterations, *h* is the PSF of the imaging system and *g* is blurred image modeled by *g* = *h* ⊗ *i*^(0)^ + *noise*^41^. The concept of this approach is illustrated in Fig. 7.

The simulated echo signal (Fig. 7(c)) is represented by convolving the PSF of the imaging system (Fig. 7(a)) with two point-targets (Fig. 7(b)). Figure 7(c,e) shows the received signal (indicated by ‘Overall’) from two targets separated by 70 μm, smaller than the spatial resolution of the system. With using deconvolution (#s of iteration = 100), however, this signal can be separated into two different targets as shown in Fig. 7(d,f). A potential problem with the RL deconvolution algorithm is noise amplification, which is known to be generic for all maximum likelihood techniques. Therefore, any false target can be expected when signal-to-noise ratio is not high enough due to noise-sensitive RL deconvolution. For example, in Fig. 7(e,f) deconvolving the received signal from two targets with significant Gaussian white noise resulted in imperfect target locations. This high sensitivity to noise can be alleviated with several available standard regulation approaches. In this study, thresholding at −10 dB was applied to pre-deconvolved signal to suppress noise component.

### Spatio-Temporal-Interframe-Correlation (STIC) based data re-alignment algorithm

**Figure 8:**
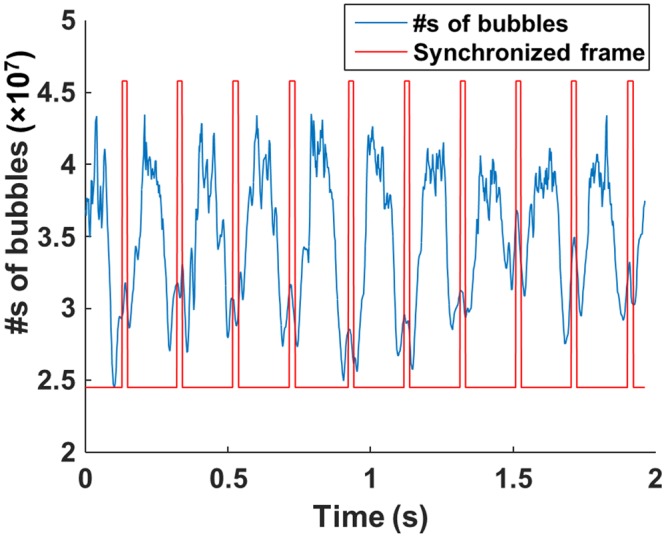
Estimated cardiac pulsation by counting the numbers of flowing microbubbles (blue solid line). After applying low-pass-filtering, the frames with minimum value are chosen as reference frames for synchronization (red solid line).

**Figure 9:**
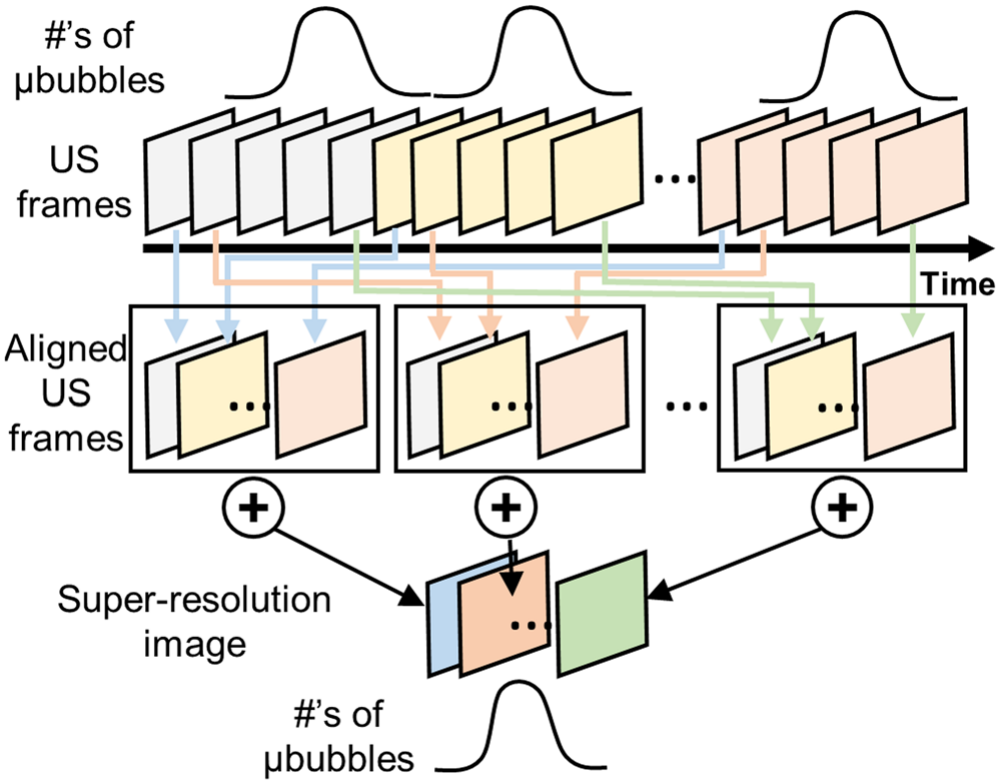
Graphical diagram of STIC data acquisition. Sequentially acquired multiple datasets are synchronized to form a single cardiac cycle event based on the cardiac pulsation estimated by the numbers of bubbles.

STIC data re-alignment algorithm that synchronizes among collected cardiac cycles based on estimated cardiac pulsation is developed to monitor fast physiological event under limited imaging speed. To capture rapid physiological dynamics at a limited frame rate, sequentially acquired RF data over multiple cardiac cycles can be synchronized based on the cardiac cycle period estimated from the periodically changing signal intensity, which reflects the number of microbubbles. Rigid motion in frame-by-frame caused by respiratory or operator dependent motion was compensated by applying an offset to match acquired images. Offset was estimated by taking 2-dimensional cross-correlation between frames. If correlation coefficient between images is smaller than 0.9, the image can be excluded. The eigen-based spatio-temporal tissue rejection filter technically removes all stationary tissue information except for moving objects, such as microbubbles. At diastolic phase, microbubbles are less likely flow, therefore smaller number of moving microbubbles are detected. On the contrary, the number of detected microbubbles significantly increases as microbubbles are moving fast toward the systole. Low-pass filter is applied to the detected signal from flowing microbubbles to estimate the cardiac period. Figure 8 illustrates an example of the number of detected microbubbles as a function of time (blue solid line) over 2 seconds totaling 1,000 data points. The reference frame for synchronization (red solid line) was chosen at the minima after low pass filtering. The estimated period of 5 Hz by this approach has good agreement with the heart rate recorded by electrocardiogram of 290–310 beats per minute. Figure 9 shows overall graphical diagram of STIC acquisition method. First, we collected raw ultrasound RF data sequentially over multiple cardiac cycles. Acquired US frames are synchronously aligned based on cardiac period estimated by the numbers of microbubbles. Re-aligned images are integrated to form a super-resolution image of single cardiac cycle. In this study, we collected continuous 3,000 frames over 6 seconds, which is equivalent to approximately 15–30 cardiac cycles. Each cardiac cycle was divided by 10 sections and entire dataset was aligned based on section number. Aligned frames in each section were summed to reconstruct single super-resolution frame. Therefore, single cardiac cycle containing 10 reconstructed frames with super-resolution was generated and each super-resolution image contains around 300 acquired frames collected over multiple cardiac cycles.

### Rabbit atherosclerotic plaque model

A New Zealand white rabbit (3.5 kg) was fed a high fat and cholesterol diet (cholesterol 1%, peanut oil 2.5%, and fat 10%) over 6 weeks to accelerate development of atherosclerosis^6^. Balloon injuries were induced to the superficial femoral artery on the right side of an anaesthetized rabbit (ketamine 150 mg IM, xylazine 8 mg IM and 2.5% inhaled isofluorane) by using a 2F Fogarty balloon catheter (Edwards Life Sciences, CA, USA) one-week after feeding. The balloon inflated at 2 atm was moved back and forth several times to apply injury to the vessel walls around bifurcation area under guidance of high-frequency ultrasound scanning (Vevo2100 equipped with a linear array transducer centered at 18.5 MHz, FUJIFILM Visualsonics, Toronto, On, Canada). Upon completion of imaging, blood of 5 mL was obtained to measure cholesterol concentration.

### Ultrasound imaging protocol

**Figure 10:**
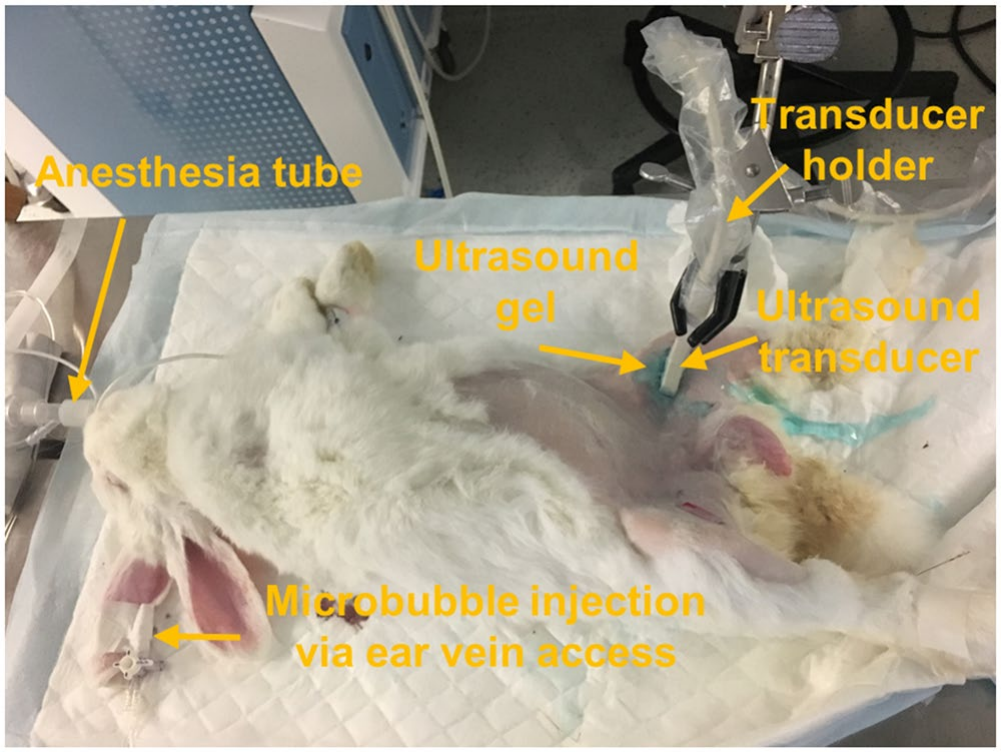
Experiment setup of rabbit imaging. Microbubbles were injected via ear vein access. A hockey stick linear array ultrasound transducer was used for imaging. Transducer holder is used to removing operator-dependent motion in this study.

At twelve weeks after surgery, ultrasound imaging were performed injecting a commercial microbubbles (Definity, Lantheus Medical Imaging, N. Billerica, MA). Mean diameter of microbubbles ranges 1.1–3.3 μm, and concentration of microbubbles is 1.2 × 10^10^ bubbles/mL. A bolus of 0.2 mL microbubbles was intravenously administrated through an ear vein access catheter for each imaging session. An anaesthetized rabbit (same protocol of surgery) was prepared with hair-shaved legs (Fig. 10). US gel was applied for ultrasonically coupling. Super-resolution US imaging sequence was performed to the surgical side and thereafter contralateral side by using a fully programmable research ultrasound scanner (Vantage 128, Verasonics, Kirkland, WI) equipped with a hockey stick linear array transducer (CL15-7, ATL-Philips, Bothell, WA). A transducer holder was used to fix the transducer. Compounded plane wave images with 3 different angles of total 9,600 frames were acquired in each data acquisition. To verify repeatability, total three datasets were collected on the possibly same imaging plane. All signal processing was performed offline using MATLAB software (Mathworks, Natick, MA). Harmonic MIP imaging was sequentially conducted on the same location by using a commercial ultrasound imaging equipped with a linear array transducer (Acuson Sequoia 512 with 15L8, SIEMENS, Mountain view, CA). To quantify degree of vasa vasorum development, vessel density was calculated by

$$Vasa\,Vasorum\,density=\frac{\sum Microbubble\,signal\,intensity\,on\,adventitia\,area}{adventitia\,area}$$

where, adventitia area was manually selected on the B-mode. The animal protocol for this study was approved by Institutional Animal Care and Use Committee (IACUC) of University of Pittsburgh. All experimental procedures of surgery and *in vivo* imaging were performed in accordance with the all guidelines and regulations detailed in the approved protocol.

### Histology and immunofluorescence

Femoral arteries were perfusion fixed using paraformaldehyde, and then carefully excised along with a block of the surrounding muscle to save neighbor vasculature in the connective tissues. Excised tissues were then carefully fixed in 4% paraformaldehyde, embedded in the paraffin-block and serially sectioned. Cross-sections of the paraffin block were stained with hematoxylin and eosin (H&E) and Von Willebrand Factor (vWF) for endothelium staining. In briefly, for antigen retrieval, treatment dewaxed slides were heated in sodium citrate buffer (pH 6.0, abcam, Cambridge, MA) at 95 °C for 30 min in blocking solution of goat serum and incubated with the anti-vWF antibody (Millipore, Burlington, MA) at 4 °C overnight. Incubated slides were washed with PBS 3 times for 3 mins. The slides were incubated with Cy3-conjugated IgG (Jackson ImmunoResearch Laboratories, West Grove, PA) and counterstained with DAPI. The stained slides were observed by using a fluorescent microscope (IX-81, Olympus, Center Valley, PA). Brightness (+40%) and contrast (+40%) of acquired fluorescence images were adjusted to improve visibility by using ImageJ software^43^.

## Electronic supplementary material

Supplementary Video1

Supplementary Video2
